# A comprehensive dataset gathering 37 cover crop field experiments across France (2004–2022): plant and soil-related agronomic variables on 33 crop species from five botanical families

**DOI:** 10.1016/j.dib.2025.111863

**Published:** 2025-07-07

**Authors:** Manon Pull, Lionel Alletto, Eric Justes, Jay Ram Lamichhane, Elana Dayoub, Guillaume Hustet-Caou, Emilie Sitnikow, Noémie Gaudio, Pierre Casadebaig, Rémi Mahmoud, Neïla Ait Kaci Ahmed, Julie Constantin, Antoine Couëdel, Noémie Deschamps, Eric Lecloux, Damien Marchand, François Perdrieux, Didier Raffaillac, Lucie Souques, Gilles Tison, Hélène Tribouillois, Alexandre Wojciechowski, Célia Seassau

**Affiliations:** a Université de Toulouse, INRAE, INPT, El PURPAN, AGIR, Castanet-Tolosan, France; b CIRAD, Persyst Department, F-34398 Montpellier, France; c UE APC, Auzeville, INRAE, France

**Keywords:** Intercropping systems, Agriculture, Ecosystem services, Functional traits, Data mining

## Abstract

Cover crops, sown between cash crops to provide ecosystem services, contribute to sustainability but present challenges related to the use of environmental resources out of the main cropping season. This dataset gathers the results of 37 field experiments related to cover crops, whether grown in sole crops or in mixtures, conducted in France over 19 years. It compiles quantitative data from field measurements and laboratory analyses on 33 species among five botanical families, along with information on technical management and climate conditions, providing a valuable resource for assessing cover crops performance.

Specifications table

**Table:** 

Subject	Agronomy and Crop Science, Soil Science
Specific subject area	Experimental dataset of cover crop experiments: management, cover crop emergence, biomass production, plant and soil nutrient content
Type of data	Raw Filtered Table
Data collection	Data were collected from experimental fields, mainly in southwest France, and include cover crop management details, field and lab measurements, and pedo-climatic data. Measurements were performed using standard agronomic and laboratory protocols. Experimental designs, materials, methods and data collection are detailed in the Experimental design, Materials, and Methods section.
Data source location	• Institution: INRAE • Location: Auzeville-Tolosane and Seysses (Haute-Garonne), Orléans (Loiret) • Country: France • Coordinates: 43.529326, 1.501011 (Auzeville-Tolosane), 43.50946, 1.251009 (Seysses), 47.776, 2.098 (Orléans)
Data accessibility	Repository name: Entrepôt Recherche Data Gouv Data identification number: doi/10.57745/3PRE07 Direct URL to data: https://doi.org/10.57745/3PRE07
Related research article	A. Couëdel, L. Alletto, H. Tribouillois, É. Justes, Cover crop crucifer-legume mixtures provide effective nitrate catch crop and nitrogen green manure ecosystem services, Agriculture, Ecosystems & Environment 254 (2018) 50–59. https://doi.org/10.1016/j.agee.2017.11.017

## 1 Value of the data

• The dataset provides a unique and extensive range of cover crop traits collected over 19 years from field experiments at research stations.
• The traits measured are categorized into soil nutrients, causes of non-emergence and post-emergence damages, plant size, development, biomass, plant nutrient and glucosinolate content.
• The cover crops, tested as sole crops or in mixtures, belong to five botanical families (Fabaceae, Brassicaceae, Poaceae, Hydrophyllaceae, Polygonaceae), with 33 species and over 128 cultivars.
• Data for each variable and modality are provided by block, depending on the experimental design. Detailed descriptions of each trial, including management practices and climate data, are supplied.
• This dataset can be used to quantify and compare cover crop traits across species and cultivars, assess cover crop performance, and to calibrate crop simulation models.

## 2 Background

Cover crops play an important role in the agroecological transition, especially in the context of climate change. In this study, we have compiled a unique dataset gathering the results of 37 field experiments in order to study the performance of several species of cover crops across differents environments {3 sites in France: Auzeville-Tolosane, Seysses and Orléans}. This dataset gathers both qualitative and quantitative data, enabling a deeper understanding of the behaviour and contributions of cover crops to sustainable practices. These data can inform methodological approaches to evaluate public policies, and support modelling and analysis of multi-performance outcomes.

## 3 Data description

This dataset gathers the results of 37 field experiments with cover crops. Cover crops are non-commercial, non-harvested plant species grown alone or in mixtures during the fallow period between successive main crops. Their primary function is to deliver multiple ecosystem services, including soil improvement, erosion control, nutrient cycling, and the management of pests and weeds.

**Fig. 1:**
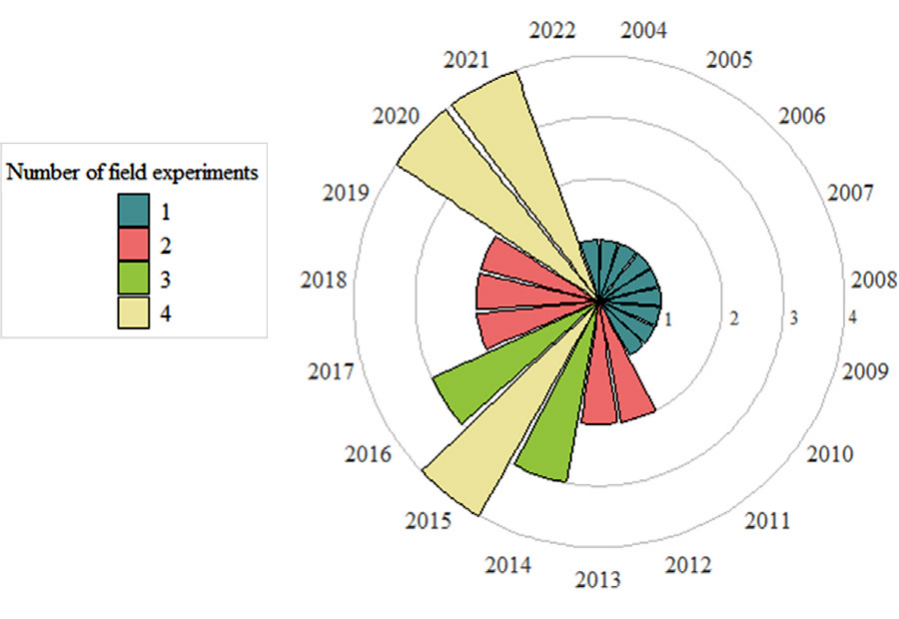
Number of experiments per year in the dataset (ranging from 1 to 4 per year).

The field experiments were carried in France, in Haute-Garonne (Auzeville-Tolosane, Seysses; *n* = 35) and in Loiret (Orléans; *n* = 2), from 2004 to 2022 (Fig. 1).

The dataset is organized as six tabular data files: three in .csv format—data_trials, data_management, and data_traits— with one observation per row and values within each row separated by a delimiter, and three in .xlsx format—data_climate, metadata, and references. The content of these files is described in Table 1, following the good practices for data tidying [1]. Files, except metadata and data_climate share a common identifier (“*experiment_id*”) which corresponds to the concatenation of “*site name-project name-year of experiment*”. Additional information on the data curation and formatting process from a raw to a tidy dataset is presented in Mahmoud et al. [2].

**Table 1:** Name and content of the six files composing the dataset.

Name	Content
metadata	Describes the table header variables (sheet dataset) and experimental measured variables with the unit of measurements (sheet variables).
data_trials	Includes the site of experiment, the project name, the year of experiment, the geographical coordinates of the fields.
data_management	This section provides information on crop management. Each entry represents a single crop cultivar, whether grown as a sole crop or within a mixture, managed in a specific experiment and year. Each cropping modality (sole crop or mixture) is associated with a specific management practice within the same experiment, labeled from M1 to Mn. Management is not necessarily continuous, as some modalities were discontinued in certain experiments (e.g., M3 in *auzeville_CIMSON_2021* and *auzeville_CIMSON_2022;* M29 in *seysses_CRUCIAL_2014).* The table includes, when available: • The genus, cultivar(s), genus mixtures, and cultivar mixtures (if applicable), along with their Latin name. • Sowing density, sowing date and termination date • Relative density (1 = full density, 0.5 = half density, etc.) • Amount of irrigation applied (if applicable) • Use of pesticides on the plot (yes/no) • Previous crop • Subsequent crop
data_climate	This dataset contains daily records of rainfall, mean/minimal/maximal temperature, and Penman-Monteith potential evapotranspiration for Auzeville-Tolosane (2004–2023) and Orléans (2014–2016), all registered by INRAE weather stations. Missing data for Auzeville-Tolosane on the following dates: 2018–04–26; 2020–06–20; 2020–12–19 to 2020–12–29; 2021–01–01; 2021–01–19.
data_traits	This dataset provides measurements conducted in each experiment at the plant, crop, or mixture level, including block-level data when applicable. The file integrates key information from the data_management with the addition of: • Measurement date • Species and species mixtures • Variable name • Block (if applicable) • Measured value
references	This section provides references to publications related to the experiments, linked to the corresponding experiment_id in the dataset.

The dataset includes 33 crop species (Table 2), among five botanical families.

**Table 2:** Summary of crop species included in the dataset.

Plant family	Species	Latin name	No. Cultivars	No. Experiments
Brassicaceae (10)	brown mustard	*Brassica juncea (*L.*) Czern*	7	16
	chinese radish	*Raphanus sativus* var. *longipinnatus* L.*H.Bailey*	2	3
	ethiopian mustard	*Brassica carinata A.Braun*	7	8
	fodder radish	*Raphanus raphanistrum* subsp. *sativus (*L.*) Domin*	7	15
	fodder rape	*Brassica napus* L.	3	4
	fodder turnip	*Brassica rapa* var. *rapa* L.	1	2
	oilseed radish	*Raphanus raphanistrum* subsp. *sativus (*L.*) Domin*	1	1
	rocket	*Eruca vesicaria* var. *sativa (Mill.) Thell.*	2	2
	turnip rape	*Brassica rapa* subsp. *oleifera (DC.) Metzg.*	5	16
	white mustard	*Sinapis alba* L.	17	29
Fabaceae (14)	bitter vetch	*Vicia ervilia (*L.*) Willd.*	1	1
	black medick	*Medicago lupulina* L.	1	1
	blue lupin	*Lupinus angustifolius* L.	1	2
	common vetch	*Vicia sativa* L.	12	15
	crimson clover	*Trifolium incarnatum* L.	3	11
	egyptian clover	*Trifolium alexandrinum* L.	3	9
	fodder lentil	*Lens nigricans (M.Bieb.) Webb & Berthel.*	1	2
	fodder pea	*Pisum sativum* L.	3	6
	hairy vetch	*Vicia villosa Brot.*	3	7
	narbonne vetch	*Vicia narbonensis* L.	2	2
	purple vetch	*Vicia benghalensis* L.	6	26
	soybean	*Glycine* max *(*L.*) Merr.*	1	4
	spring fababean	*Vicia faba* L.	3	6
	winter fababean	*Vicia faba* L.	7	13
Hydrophyllaceae (1)	phacelia	*Phacelia tanacetifolia Benth.*	3	10
Poaceae (7)	black oat	*Avena strigosa Schreb.*	9	11
	fodder sorghum	*Sorghum bicolor (*L.*) Moench*	1	3
		*Sorghum bicolor (*L.*) Moench x Sorghum bicolor nothosubsp. drummondii (Nees ex Steud.) de Wet ex Davidse*	1	1
	forest rye	*Secale cereale* L.	3	5
	italian ryegrass	*Lolium multiflorum Lam.*	1	2
	moha	*Setaria italica* subsp. *moharia (Alef.) H.Scholz*	2	8
	sorghum sudangrass	*Sorghum bicolor nothosubsp. drummondii (Nees ex Steud) de Wet ex Davidse*	1	5
	spring oat	*Avena sativa* L.	3	7
Polygonaceae (1)	buckwheat	*Fagopyrum esculentum Moench*	2	2

Each species can be present in one or more experiments. Each experiment corresponds to a unique {site * year} combination and includes one or more cultivars.

Some experiments include species mixtures, with 2 to 4 species grown simultaneously. This results in 10 types of intercropping based on botanical family mixtures (Table 3).

**Table 3:** Summary of cover crops mixtures included in the dataset.

Plant family mixtures	Species mixtures	No. Species	No. Experiments
Brassicaceae (1)	turnip rape-white mustard	2	1
Brassicaceae-Fabaceae (52)	brown mustard-common vetch	2	4
	brown mustard-egyptian clover	2	3
	brown mustard-fodder radish-purple vetch	3	3
	brown mustard-purple vetch	2	5
	brown mustard-spring fababean	2	2
	chinese radish-spring fababean	2	2
	ethiopian mustard-common vetch	2	5
	ethiopian mustard-egyptian clover	2	2
	ethiopian mustard-hairy vetch	2	2
	ethiopian mustard-purple vetch	2	3
	ethiopian mustard-soybean	2	2
	ethiopian mustard-winter fababean	2	1
	fodder radish-blue lupin	2	1
	fodder radish-common vetch	2	2
	fodder radish-egyptian clover	2	5
	fodder radish-hairy vetch	2	4
	fodder radish-purple vetch	2	6
	fodder radish-turnip rape-purple vetch	3	3
	fodder radish-white mustard-hairy vetch	3	2
	fodder rape-blue lupin	2	1
	fodder rape-common vetch	2	4
	fodder rape-crimson clover	2	2
	fodder rape-egyptian clover	2	4
	fodder rape-hairy vetch	2	2
	fodder rape-narbonne vetch	2	1
	fodder rape-purple vetch	2	4
	fodder rape-soybean	2	2
	fodder rape-winter fababean	2	2
	oilseed radish-egyptian clover	2	1
	oilseed radish-hairy vetch	2	1
	rocket-common vetch	2	1
	rocket-crimson clover	2	1
	rocket-egyptian clover	2	1
	rocket-purple vetch	2	1
	turnip rape-black medick	2	1
	turnip rape-common vetch	2	2
	turnip rape-crimson clover	2	1
	turnip rape-egyptian clover	2	3
	turnip rape-fodder lentil	2	1
	turnip rape-fodder pea	2	1
	turnip rape-purple vetch	2	8
	turnip rape-spring fababean	2	2
	turnip rape-winter fababean	2	2
	white mustard-blue lupin	2	1
	white mustard-common vetch	2	4
	white mustard-egyptian clover	2	3
	white mustard-fodder pea	2	1
	white mustard-hairy vetch	2	2
	white mustard-narbonne vetch	2	1
	white mustard-purple vetch	2	9
	white mustard-soybean	2	2
	white mustard-winter fababean	2	2
Brassicaceae-Fabaceae-Hydrophyllaceae (3)	brown mustard-winter fababean-phacelia	3	2
	chinese radish-common purple vetch-spring fababean-phacelia	4	1
	turnip rape-purple vetch-spring fababean-phacelia	4	1
Brassicaceae-Fabaceae-Poaceae (1)	white mustard-crimson clover-winter fababean-moha	4	1
Brassicaceae-Poaceae (4)	turnip rape-moha-sorghum sudangrass	3	1
	white mustard-fodder sorghum	2	2
	white mustard-moha	2	3
	white mustard-sorghum sudangrass	2	1
Fabaceae (6)	common vetch-fodder pea	2	1
	crimson clover-purple vetch	2	1
	fodder pea-purple vetch	2	2
	fodder turnip-common vetch	2	1
	fodder turnip-egyptian clover	2	1
	fodder turnip-purple vetch	2	1
Fabaceae-Hydrophyllaceae (8)	black medick-phacelia	2	1
	common vetch-spring fababean-phacelia	3	1
	crimson clover-phacelia	2	2
	fodder lentil-phacelia	2	1
	fodder pea-phacelia	2	1
	purple vetch-phacelia	2	4
	spring fababean-phacelia	2	2
	winter fababean-phacelia	2	1
Fabaceae-Hydrophyllaceae-Poaceae (2)	spring fababean-phacelia-forest rye	3	1
	spring fababean-phacelia-forest rye-sorghum sudangrass	4	1
Fabaceae-Poaceae (28)	black medick-black oat	2	1
	black medick-italian ryegrass	2	1
	black medick-moha	2	1
	common vetch-moha	2	1
	common vetch-sorghum sudangrass	2	1
	common vetch-spring oat	2	6
	crimson clover-black oat	2	1
	crimson clover-fodder sorghum	2	1
	crimson clover-italian ryegrass	2	2
	crimson clover-moha	2	2
	crimson clover-winter fababean-black oat	3	3
	egyptian clover-fodder sorghum	2	1
	egyptian clover-purple vetch-moha-sorghum sudangrass	4	1
	egyptian clover-purple vetch-sorghum sudangrass	3	1
	fodder lentil-black oat	2	1
	fodder lentil-italian ryegrass	2	1
	fodder lentil-moha	2	1
	fodder pea-black oat	2	2
	fodder pea-italian ryegrass	2	2
	fodder pea-moha	2	1
	fodder pea-purple vetch-forest rye	3	2
	purple vetch-black oat	2	10
	purple vetch-italian ryegrass	2	1
	purple vetch-moha	2	2
	spring fababean-black oat	2	1
	spring fababean-fodder sorghum	2	1
	spring fababean-italian ryegrass	2	1
	spring fababean-moha	2	2
Hydrophyllaceae-Poaceae (1)	phacelia-black oat	2	3

**Fig. 2:**
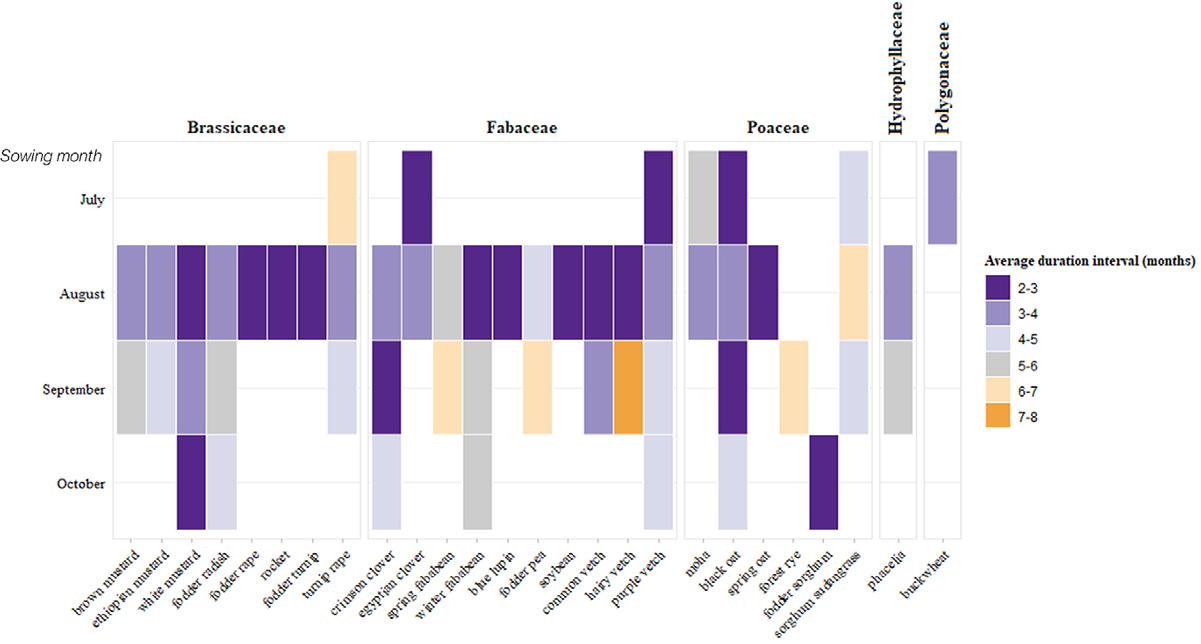
Average duration of cover crops by species and sowing month. Species are grouped by botanical family: Brassicaceae, Fabaceae, Poaceae, Hydrophyllaceae and Polygonaceae.

The dataset includes data on cover crops sown at various times throughout the growing season and for different durations (Table 4). Within the same experiment, sowing and termination dates may vary. For further details, Fig. 2 presents the average duration of the cover crops per species according to the month of sowing. Only species present in at least two experiments are shown.

**Table 4:** Overview of cover crops sowing period and average duration across experiments.

Sowing month	No. Experiments	Average duration (months) of cover crops from sowing to termination ± sd
July	5	4.2 ± 2
August	21	3.1 ± 1.3
September	21	3.3 ± 1.7
October	7	3.9 ± 1.2
November	1	5.5

Pooling the 37 field experiments, a total of 141 variables were measured, though not systematically across all experiments. Table 5 provides an overview of these variables along with the number of experiments in which each was assessed.

**Table 5:** Summary of measured variables categorized by soil nutrients, causes of non-emergence and post-emergence damages, plant size, development and content, biomass, plant nutrient content, and glucosinolate content. Each variable is presented with its unit and the number of experiments (No. Experiments) in which it was measured. Categories are organized chronologically according to the timing of the measurements, with variables listed alphabetically within each category.

Category	Variable	Unit	No. Experiments
Soil	soil_nitrogen_0–30	kg.ha^-1^	35
Soil	soil_nitrogen_30–60	kg.ha^-1^	35
Soil	soil_nitrogen_60–90	kg.ha^-1^	34
Soil	soil_nitrogen_90–120	kg.ha^-1^	25
Soil	soil_sulphur_0–30	kg.ha^-1^	6
Soil	soil_sulphur_30–60	kg.ha^-1^	6
Soil	soil_sulphur_60–90	kg.ha^-1^	6
Soil	soil_sulphur_90–120	kg.ha^-1^	1
Soil	soil_swc_0–30	mm	22
Soil	soil_swc_30–60	mm	22
Soil	soil_swc_60–90	mm	22
Soil	soil_swc_90–120	mm	15
Causes of non-emergence and post-emergence damages	CNE_abiotic_stress	–	2
Causes of non-emergence and post-emergence damages	CNE_clods	–	2
Causes of non-emergence and post-emergence damages	CNE_crusting	–	1
Causes of non-emergence and post-emergence damages	CNE_damping_off	–	1
Causes of non-emergence and post-emergence damages	CNE_land_pests	–	1
Causes of non-emergence and post-emergence damages	CNE_seedlessness_birds	–	2
Causes of non-emergence and post-emergence damages	CNE_sowing_depth	–	2
Causes of non-emergence and post-emergence damages	PED_animals	–	2
Causes of non-emergence and post-emergence damages	PED_anoxia	–	1
Causes of non-emergence and post-emergence damages	PED_avifauna	–	1
Causes of non-emergence and post-emergence damages	PED_damping_off	–	1
Causes of non-emergence and post-emergence damages	PED_flea_beetles	–	2
Causes of non-emergence and post-emergence damages	PED_freeze	–	1
Causes of non-emergence and post-emergence damages	PED_land_pests	–	1
Causes of non-emergence and post-emergence damages	PED_slugs	–	2
Causes of non-emergence and post-emergence damages	PED_thermal_stress	–	1
Plant size, development and content	adf_shoot	%	4
Plant size, development and content	adl_shoot	%	4
Plant size, development and content	cellulose_shoot	%	4
Plant size, development and content	cover	%	5
Plant size, development and content	emergence_density	plant.m^-2^	15
Plant size, development and content	height	cm	5
Plant size, development and content	hemicellulose_shoot	%	4
Plant size, development and content	leaf_area	cm²	2
Plant size, development and content	leaf_number	–	1
Plant size, development and content	length	cm	2
Plant size, development and content	ndf_shoot	%	4
Plant size, development and content	stage	–	5
Biomass	biomass_root	t.ha^-1^ of dry matter	26
Biomass	biomass_root_regrowth_previous_crop	t.ha^-1^ of dry matter	1
Biomass	biomass_shoot	t.ha^-1^ of dry matter	36
Biomass	biomass_shoot_regrowth_previous_crop	t.ha^-1^ of dry matter	3
Biomass	biomass_shoot_weed	t.ha^-1^ of dry matter	18
Biomass	humidity_root	%	2
Biomass	humidity_shoot	%	9
Biomass	humidity_shoot_weed	%	3
Biomass	root_shoot_ratio	–	2
Plant nutrient content	boron_root	mg.kg^-1^	1
Plant nutrient content	boron_shoot	mg.kg^-1^	1
Plant nutrient content	calcium_root	%	1
Plant nutrient content	calcium_shoot	%	1
Plant nutrient content	carbon_acq_root	kg.ha^-1^	16
Plant nutrient content	carbon_acq_root_regrowth_previous_crop	kg.ha^-1^	1
Plant nutrient content	carbon_acq_shoot	kg.ha^-1^	27
Plant nutrient content	carbon_acq_shoot_regrowth_previous_crop	kg.ha^-1^	1
Plant nutrient content	carbon_acq_shoot_weed	kg.ha^-1^	9
Plant nutrient content	carbon_root	%	17
Plant nutrient content	carbon_root_regrowth_previous_crop	%	1
Plant nutrient content	carbon_shoot	%	28
Plant nutrient content	carbon_shoot_regrowth_previous_crop	%	1
Plant nutrient content	carbon_shoot_weed	%	10
Plant nutrient content	copper_root	mg.kg^-1^	1
Plant nutrient content	copper_shoot	mg.kg^-1^	1
Plant nutrient content	delta_15N_root	–	3
Plant nutrient content	delta_15N_shoot	–	18
Plant nutrient content	delta_15N_shoot_regrowth_previous_crop	–	3
Plant nutrient content	delta_15N_shoot_weed	–	6
Plant nutrient content	iron_root	mg.kg^-1^	1
Plant nutrient content	iron_shoot	mg.kg^-1^	1
Plant nutrient content	magnesium_root	%	1
Plant nutrient content	magnesium_shoot	%	1
Plant nutrient content	manganese_root	mg.kg^-1^	1
Plant nutrient content	manganese_shoot	mg.kg^-1^	1
Plant nutrient content	nitrogen_acq_root	kg.ha^-1^	25
Plant nutrient content	nitrogen_acq_root_regrowth_previous_crop	kg.ha^-1^	1
Plant nutrient content	nitrogen_acq_shoot	kg.ha^-1^	36
Plant nutrient content	nitrogen_acq_shoot_regrowth_previous_crop	kg.ha^-1^	3
Plant nutrient content	nitrogen_acq_shoot_weed	kg.ha^-1^	13
Plant nutrient content	nitrogen_root	%	28
Plant nutrient content	nitrogen_root_regrowth_previous_crop	%	1
Plant nutrient content	nitrogen_shoot	%	37
Plant nutrient content	nitrogen_shoot_regrowth_previous_crop	%	3
Plant nutrient content	nitrogen_shoot_weed	%	14
Plant nutrient content	phosphorus_acq_shoot	kg.ha^-1^	1
Plant nutrient content	phosphorus_root	%	1
Plant nutrient content	phosphorus_shoot	%	2
Plant nutrient content	potassium_root	%	1
Plant nutrient content	potassium_shoot	%	1
Plant nutrient content	sodium_root	%	1
Plant nutrient content	sodium_shoot	%	1
Plant nutrient content	sulphur_acq_root	kg.ha^-1^	9
Plant nutrient content	sulphur_acq_shoot	kg.ha^-1^	12
Plant nutrient content	sulphur_root	%	11
Plant nutrient content	sulphur_shoot	%	14
Plant nutrient content	sulphur_shoot_weed	%	1
Plant nutrient content	zinc_root	mg.kg^-1^	1
Plant nutrient content	zinc_shoot	mg.kg^-1^	1
Glucosinolate content	4hydroxyglucobrassicin_root	µmol.g^-1^ of dry matter	9
Glucosinolate content	4hydroxyglucobrassicin_shoot	µmol.g^-1^ of dry matter	9
Glucosinolate content	4methoxyglucobrassicin_root	µmol.g^-1^ of dry matter	9
Glucosinolate content	4methoxyglucobrassicin_shoot	µmol.g^-1^ of dry matter	9
Glucosinolate content	epiprogoitrin_root	µmol.g^-1^ of dry matter	2
Glucosinolate content	epiprogoitrin_shoot	µmol.g^-1^ of dry matter	2
Glucosinolate content	glucoalyssin_root	µmol.g^-1^ of dry matter	5
Glucosinolate content	glucoalyssin_shoot	µmol.g^-1^ of dry matter	4
Glucosinolate content	glucobrassicanapin_root	µmol.g^-1^ of dry matter	8
Glucosinolate content	glucobrassicanapin_shoot	µmol.g^-1^ of dry matter	7
Glucosinolate content	glucobrassicin_root	µmol.g^-1^ of dry matter	9
Glucosinolate content	glucobrassicin_shoot	µmol.g^-1^ of dry matter	9
Glucosinolate content	glucoerucin_root	µmol.g^-1^ of dry matter	9
Glucosinolate content	glucoerucin_shoot	µmol.g^-1^ of dry matter	9
Glucosinolate content	glucoiberin_root	µmol.g^-1^ of dry matter	2
Glucosinolate content	glucoiberin_shoot	µmol.g^-1^ of dry matter	2
Glucosinolate content	gluconapin_root	µmol.g^-1^ of dry matter	9
Glucosinolate content	gluconapin_shoot	µmol.g^-1^ of dry matter	9
Glucosinolate content	gluconapoleiferin_root	µmol.g^-1^ of dry matter	7
Glucosinolate content	gluconapoleiferin_shoot	µmol.g^-1^ of dry matter	7
Glucosinolate content	gluconasturtiin_root	µmol.g^-1^ of dry matter	9
Glucosinolate content	gluconasturtiin_shoot	µmol.g^-1^ of dry matter	9
Glucosinolate content	glucoraphanin_root	µmol.g^-1^ of dry matter	9
Glucosinolate content	glucoraphanin_shoot	µmol.g^-1^ of dry matter	9
Glucosinolate content	glucoraphasatin_E/Z_mixture_II_root	µmol.g^-1^ of dry matter	2
Glucosinolate content	glucoraphasatin_E/Z_mixture_II_shoot	µmol.g^-1^ of dry matter	2
Glucosinolate content	glucoraphasatin_E/Z_mixture_I_root	µmol.g^-1^ of dry matter	2
Glucosinolate content	glucoraphasatin_E/Z_mixture_I_shoot	µmol.g^-1^ of dry matter	2
Glucosinolate content	glucoraphasatin_root	µmol.g^-1^ of dry matter	1
Glucosinolate content	glucoraphasatin_shoot	µmol.g^-1^ of dry matter	1
Glucosinolate content	glucoraphenin_root	µmol.g^-1^ of dry matter	2
Glucosinolate content	glucoraphenin_shoot	µmol.g^-1^ of dry matter	2
Glucosinolate content	glucotropaeolin_root	µmol.g^-1^ of dry matter	7
Glucosinolate content	glucotropaeolin_shoot	µmol.g^-1^ of dry matter	7
Glucosinolate content	neoglucobrassicin_root	µmol.g^-1^ of dry matter	9
Glucosinolate content	neoglucobrassicin_shoot	µmol.g^-1^ of dry matter	9
Glucosinolate content	progoitrin_root	µmol.g^-1^ of dry matter	9
Glucosinolate content	progoitrin_shoot	µmol.g^-1^ of dry matter	9
Glucosinolate content	sinalbin_root	µmol.g^-1^ of dry matter	7
Glucosinolate content	sinalbin_shoot	µmol.g^-1^ of dry matter	7
Glucosinolate content	sinigrin_root	µmol.g^-1^ of dry matter	9
Glucosinolate content	sinigrin_shoot	µmol.g^-1^ of dry matter	9

**Fig. 3:**
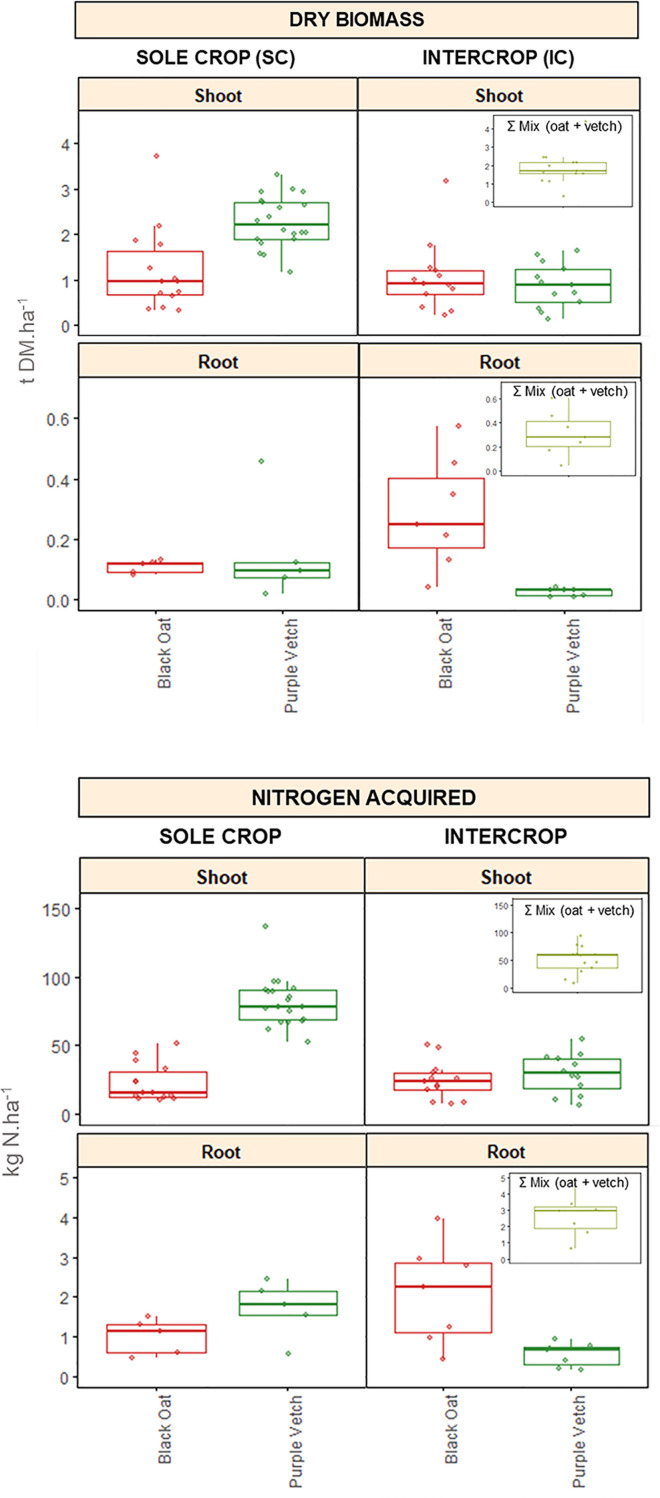

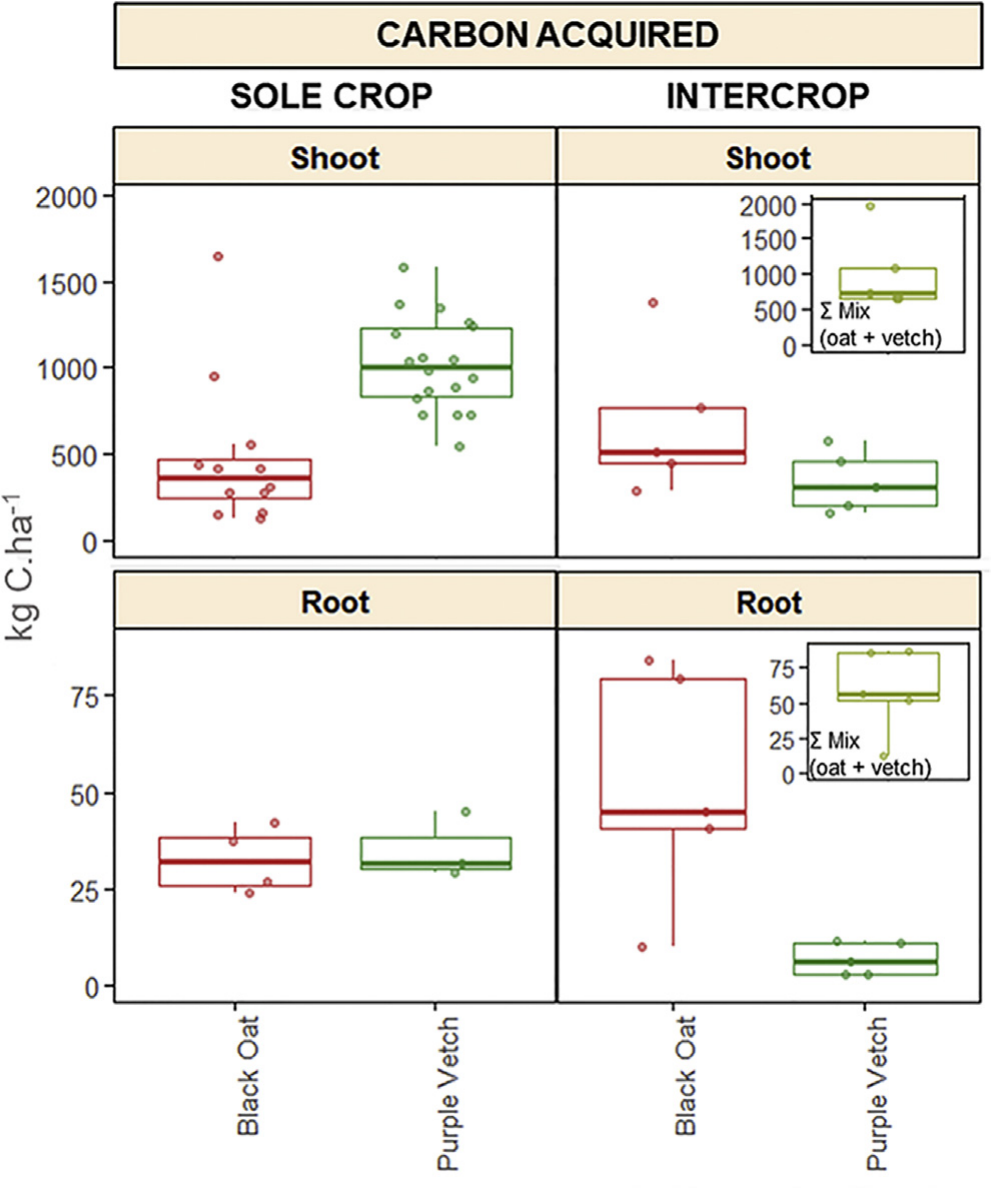
Biomass, nitrogen and carbon uptake by shoots and roots of black oat and purple vetch grown as sole crop or mixture. SC = measurements for black oat and purple vetch in sole crops; IC = separate measurements for black oat and purple vetch in intercrop; Mix (oat + vetch) = sum of both species in the mixture.

To illustrate key features from the dataset, we focused on i) the most represented species mixture, i.e. oat-vetch (*n* = 10) and ii) variables that were most frequently measured across experiments i.e. biomass production (*biomass_shoot, biomass_root : t.ha^-1^ of dry matter*), nitrogen and carbon uptake by plants (*carbon_acq_shoot, carbon_acq_root, nitrogen_acq_shoot, nitrogen_acq_root : kg.ha^-1^*) (Fig. 3). Shoot and root biomass data were not available for all modalities. Consequently, the shoot and root boxplots are independent and based on different sample sizes. However, whenever biomass shoot is available, biomass root is also present, but the reverse is not always true.

## 4 Experimental design, materials and methods

### 4.1 Experimental site and design

The experiments included in the database were conducted between 2004 and 2022 at three sites: the INRAE research station of Auzeville-Tolosane (43.529326, 1.501011), the Lamothe experimental farm of INP-EI Purpan located at Seysses (43.50946, 1.251009), and a private research station near Orléans (47.776, 2.098).

The experiments were carried out in experimental fields though two types of trials: analytical and cropping system trials. In the analytical trials, cover crops were sown in microplots with a minimum size of 14 m², while in the cropping system trials, cover crops were sown in strips, each covering at least 3000 m². The sites, number of crop campaigns, experimental design and experiment identification are summarized in Table 6, [3], [4], [5], [6], [7], [8], [9], [10], [11], [12], [13], [14].

**Table 6:** Summary of the database experiments including site, number of campaigns, experimental design and the corresponding experiment_id linked to each experiment.

The management and features described refer to the main features of the experiments, or specific parameters related to certain experiments. Detailed management-specific data are detailed in the *data_management* table.

### 4.2 Cover crops management

Before sowing, shallow tillage was carried out for all cover crops except for two managements. Irrigation was applied at sowing when necessary (19 out of 37 experiments) to ensure uniform emergence and robust plant establishment. Cover crops were not directly fertilized; however, in some cropping system trials, plots received phosphorus (P) fertilization during or just before the sowing of the cover crop to meet the nutrient requirements for subsequent cash crops.

Only a few experiments (8 out of 37) received plant protection treatments during the cover crop growth phase. These treatments were mainly limited to pesticides against slugs, insecticides targeting specific pests such as sawfly, or herbicides for weed control. Some experiments included bare soil as a control treatment, most of them being kept weed-free using mechanical methods.

### 4.3 Measurement and sampling

Table 7 provides a description of the methods.

**Table 7:** Summary of the method descriptions by variable category. Categories are organized chronologically according to the timing of the measurements, with variables listed alphabetically within each category and comparable method grouping.

Category	Variable	Method description
Soil	soil_nitrogen_0–30 soil_nitrogen_30–60 soil_nitrogen_60–90 soil_nitrogen_90–120 soil_sulphur_0–30 soil_sulphur_30–60 soil_sulphur_60–90 soil_sulphur_90–120 soil_swc_0–30 soil_swc_30–60 soil_swc_60–90 soil_swc_90–120	Depending on the experiments, soil samples were collected both before sowing and at the end of the growing season (*n* = 9), or only at the cover crop termination (*n* = 26). Soil cores were randomly extracted using a hydraulic core drill at different horizons with depths varying according to the experiment (0–30, 30–60, 60–90, 90–120 cm). Soil mineral nitrogen (nitrate and ammonium) and soil sulfur concentrations were analyzed using a continuous flow auto-analyzer (Skylar 51,000, Skalar Analytic, Breda, Netherlands) following the standard NF ISO 14,256–2. Soil water content was calculated by multiplying the measured gravimetric water content by the estimated bulk density.
Causes of non-emergence and post-emergence damages	CNE_abiotic_stress CNE_clods CNE_crusting CNE_damping_off CNE_land_pests CNE_seedlessness_birds CNE_sowing_depth PED_animals PED_anoxia PED_avifauna PED_damping_off PED_flea_beetles PED_freeze PED_land_pests PED_slugs PED_thermal_stress	During the emergence phase, the causes of non-emergence were identified at a set number of empty points within subplots, using a visual diagnostic key [15]. At each empty point, once seeds or seedling remnants were found the likely causes of non-emergence were recorded. For each subplot, the percentage of occurrences attributed to each identified cause was calculated. The same approach was applied during the post-emergence phase to assess damage occurring after seedling emergence. Plants with damage were counted and the percentage of each type of damage relative to the total number of plants that had emerged was calculated.
Plant size, development and content	adl_shoot adf_shoot cellulose_shoot hemicellulose_shoot ndf_shoot	Some plant samples were analyzed in a specialized laboratory to determine cell wall components using the Van Soest method [16], with results expressed as cellulose, hemicellulose, and acid detergent lignin (ADL), acid detergent fiber (ADF), and neutral detergent fiber (NDF) content.
	cover	The soil cover provided by the cover crops was assessed through visual observation and scored on a scale from 1 to 5, where 1 corresponded to 0–12.5 % cover, 2 to 12.5–25 %, 3 to 25–50 %, 4 to 50–75 % and 5 corresponded to 75–100 % cover. In the database, these results are expressed as a percentage.
	height leaf_area leaf_number length	Plant traits were measured or counted on selected plants within plots or subplots, including height, length, and leaf number. Sampled plants were collected for leaf area measurements using a planimeter (LI-COR 3100C) or a manual scanner, these methods are comparable. The reported plant height and length corresponded to the aerial part only (shoot).
	emergence_density	A few days after sowing, the number of emerged plants for each cover crop species was counted within areas measuring 0.5 to 1 m². The results were then expressed as plants per square meter (plants.m^-2^).
	stage	Visual observation (BBCH scale).
Biomass	biomass_root biomass_root_regrowth_previous_crop biomass_shoot biomass_shoot_regrowth_previous_crop biomass_shoot_weed humidity_root humidity_shoot humidity_shoot_weed root_shoot_ratio	To estimate biomass produced by cover crops, because of the sampling method the estimated biomass include weeds, or regrowth from previous crops, shoot biomass samples were collected for each species at cover crop termination using areas of 0.25 m² to 1 m². In some experiments, root biomass was collected, typically at a depth from 25–30 cm, either for all species or selected ones, and from either all subplots, specific zones, or a subset of plants. This was done to calculate the root:shoot ratio. Fresh shoot and root samples were weighed, sub-sampled, dried at 80 °C for 48 hours, and then reweighed to estimate dry matter biomass per species.
Plant nutrient content	boron_root boron_shoot calcium_root calcium_shoot carbon_acq_root carbon_acq_root_regrowth_previous_crop carbon_acq_shoot carbon_acq_shoot_regrowth_previous_crop carbon_acq_shoot_weed carbon_root carbon_root_regrowth_previous_crop carbon_shoot carbon_shoot_regrowth_previous_crop carbon_shoot_weed copper_root copper_shoot iron_root iron_shoot magnesium_root magnesium_shoot manganese_root manganese_shoot nitrogen_acq_root nitrogen_acq_root_regrowth_previous_crop nitrogen_acq_shoot nitrogen_acq_shoot_regrowth_previous_crop nitrogen_acq_shoot_weed nitrogen_root nitrogen_root_regrowth_previous_crop nitrogen_shoot nitrogen_shoot_regrowth_previous_crop nitrogen_shoot_weed phosphorus_acq_shoot phosphorus_root phosphorus_shoot potassium_root potassium_shoot sodium_root sodium_shoot sulphur_acq_root sulphur_acq_shoot sulphur_root sulphur_shoot sulphur_shoot_weed zinc_root zinc_shoot	Dried biomass samples were ground and analyzed for nitrogen (N), carbon (C), sulphur (S) or other mineral element concentrations using the Dumas method (MicroVario Cube, Elementar, Langenselbold, Germany).
	delta_15N_root delta_15N_shoot delta_15N_shoot_regrowth_previous_crop delta_15N_shoot_weed	In some experiments, 15 N concentration was measured using a stable isotope ratio mass spectrometer in continuous flow (Isoprime 100, UK) to determine the percentage of plant nitrogen (N) derived from the atmosphere.
Glucosinolate content	4hydroxyglucobrassicin_root 4hydroxyglucobrassicin_shoot 4methoxyglucobrassicin_root 4methoxyglucobrassicin_shoot epiprogoitrin_root epiprogoitrin_shoot glucoalyssin_root glucoalyssin_shoot glucobrassicanapin_root glucobrassicanapin_shoot glucobrassicin_root glucobrassicin_shoot glucoerucin_root glucoerucin_shoot glucoiberin_root glucoiberin_shoot gluconapin_root gluconapin_shoot gluconapoleiferin_root gluconapoleiferin_shoot gluconasturtiin_root gluconasturtiin_shoot glucoraphanin_root glucoraphanin_shoot glucoraphasatin_E/Z_mixture_II_root glucoraphasatin_E/Z_mixture_II_shoot glucoraphasatin_E/Z_mixture_I_root glucoraphasatin_E/Z_mixture_I_shoot glucoraphasatin_root glucoraphasatin_shoot glucoraphenin_root glucoraphenin_shoot glucotropaeolin_root glucotropaeolin_shoot neoglucobrassicin_root neoglucobrassicin_shoot progoitrin_root progoitrin_shoot sinalbin_root sinalbin_shoot sinigrin_root sinigrin_shoot	For glucosinolate analysis, a sub-sample of Brassicaceae plants were collected per plot, cut, and frozen at −80 °C before lyophilization. Glucosinolate profiles and concentrations were then analyzed according to their characteristics as described by de Graaf et al. [17].

## 5 Limitations

The data originate from a variety of experiments conducted on different plots, which introduces a certain degree of pedological variability, despite the relative geographical proximity of the sites. Some level of heterogeneity is also expected due to variations in experimental designs, the types of phenotypic traits recorded, the organizational scale of measurements (organ, plant, crop), and the completeness of available data. In some cases, not all variables were measured across all experiments, which may slightly limit the potential for direct comparisons. Additionally, a few management data are missing. When exact measurement dates were unavailable, a reasonable approximation was made by assigning a date close to the known cover crop termination period.

## 6 Ethics statement

All participants provided informed consent prior to their inclusion in the study. The study was approved by the appropriate institutional research ethics committees and this dataset does not involve human subjects, animal experiments, or sensitive data.

## CRediT authorship contribution statement

**Manon Pull:** Data curation, Writing – original draft, Writing – review & editing. **Lionel Alletto:** Conceptualization, Funding acquisition, Investigation, Writing – review & editing. **Eric Justes:** Conceptualization, Investigation, Funding acquisition, Writing – review & editing. **Jay Ram Lamichhane:** Conceptualization, Investigation, Writing – review & editing. **Elana Dayoub:** Data curation. **Guillaume Hustet-Caou:** Data curation. **Emilie Sitnikow:** Data curation. **Noémie Gaudio:** Conceptualization, Data curation, Methodology, Software, Writing – review & editing. **Pierre Casadebaig:** Conceptualization, Methodology, Software, Writing – review & editing. **Rémi Mahmoud:** Conceptualization, Methodology, Software. **Neïla Ait Kaci Ahmed:** Investigation. **Julie Constantin:** Conceptualization, Investigation. **Antoine Couëdel:** Investigation. **Noémie Deschamps:** Investigation. **Eric Lecloux:** Investigation. **Damien Marchand:** Investigation. **François Perdrieux:** Investigation. **Didier Raffaillac:** Investigation. **Lucie Souques:** Investigation. **Gilles Tison:** Investigation. **Hélène Tribouillois:** Investigation. **Alexandre Wojciechowski:** Investigation. **Célia Seassau:** Conceptualization, Funding acquisition, Investigation, Writing – review & editing.

## Data Availability

https://entrepot.recherche.data.gouv.frA comprehensive dataset gathering 37 cover crop field experiments across France (2004–2022): plant and soil-related agronomic variables on 33 crop species from five botanical families
